# Descriptions of a new genus and a new species, *Grylloprimevala jilina* (Grylloblattidae) from China

**DOI:** 10.1002/ece3.9750

**Published:** 2023-01-19

**Authors:** Lin Zhou, Qi Chen, Haoqin Ke, Zizhuo Wang, Jie Peng, Donghui Wu, Ying Liu, Jiang Feng, Bingzhong Ren

**Affiliations:** ^1^ Jilin Provincial Key Laboratory of Animal Resource Conservation and Utilization Northeast Normal University Changchun China; ^2^ Key Laboratory of Vegetation Ecology, School of Environment Institute of Grassland Science, Northeast Normal University, Ministry of Education Changchun China; ^3^ College of Life Science Jilin Agricultural University Changchun China

**Keywords:** diversity, Grylloblattidae, Grylloblattodea, morphology, new species, phylogeny

## Abstract

We erected a new genus (*Grylloprimevala* Zhou & Ren gen. nov.) and defined a new species (*Grylloprimevala jilina* Zhou & Ren sp. nov.) from a natural cave in the primeval forest of Jilin Province, China, according to the morphological, behavioral, and molecular evidence. *Grylloprimevala* gen. nov. is distinguishable from other genera of Grylloblattodea primarily by morphological characters, including the slightly concave posterior margin of the pronotum and no poorly sclerotized zones, six intramarginal and nine intermarginal setae on the cervical sclerite, one tooth on the lacinia, no pulvilli on tarsal segments, and a symmetrical epiproct with a pointed triangular and middle‐depressed median projection on the posterior margin. Based on the morphological features mentioned above, we further identified a new species, *G. jilina* sp. nov. At the aspect of behavior, *G. jilina* sp. nov. displays the typical characteristics of troglobites, including degraded visual senses, developed body surface sensors, and predation between individuals. Furthermore, molecular phylogenetic analyses also supported the morphological delimitation of *G. jilina* sp. nov. due to the separate clade of *G. jilina* sp. nov. Our results provide materials for the determination and conservation of Grylloblattodea in China.

## Graphical abstract

A new species, *Grylloprimevala jilina* Zhou & Ren sp. nov. assigned to a new genus *Grylloprimevala* Zhou & Ren gen. nov. (Grylloblattodea), is described and defined using specimens from a natural cave in the primeval forest of Jilin Province, China. We integrated morphological, behavioral, and molecular evidence to describe the new species and genus. Our results provide materials for the determination and conservation of Grylloblattodea in China.

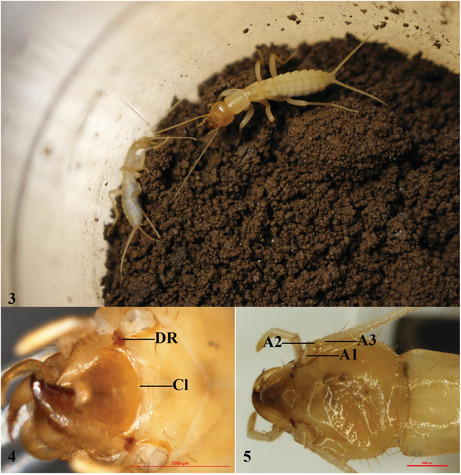

## 1 INTRODUCTION

Grylloblattodea is known as the only “living fossil” of insects (Crampton, 1926; Walker, 1937), retaining primitive features of Orthoptera along with features of Plecoptera, Embiodea, and Thysanura (Wang, 1999). As the only relict order of Insecta, Grylloblattodea is associated with the extinct Protoblattoidea and may be more closely related to Protorthoptera (Bai et al., 2010; Rasnitsyn, 2002). According to the studies on the distribution of fossil and extant families, Grylloblattodea are intermediates in the course of evolution from winged to wingless phenotypes and from ubiquitous to restricted distributions, and they represent a rare order on the verge of extinction (Storozhenko & Park, 2002; Terry & Whiting, 2005). Grylloblattodea contains 47 families, including 46 fossil families (from the Late Carboniferous period) and Grylloblattidae (the common name: icebugs/ice crawlers) that contains all extant 40 species and subspecies in five genera (*Grylloblatta*, *Grylloblattina*, *Galloisiana*, *Namkungia*, and *Grylloblattella*) (Aristov & Zessin, 2009; Bai et al., 2010; Storozhenko, 1992, 1997). However, the relationships between Grylloblattidae and the fossil families remain unclear (Eberhard et al., 2018). Therefore, the study of Grylloblattidae is of significant for exploring the origin and evolution of insects with the succession of geological history (Cui et al., 2019).

Grylloblattidae have many primitive features, including chewing mouthparts with a prognathous head, three movable thoracic segments with the original muscle connection, males having well‐developed asymmetrical abdominal coxites at the ninth segment with apical styli. The morphology of Grylloblattodea insects has remained relatively stable during 300 million years of evolution with only minimal changes, and the most significant difference between extant and extinct Grylloblattidae is found in the thoracic segments (Misof et al., 2014). The thorax containing the muscles of legs and wings has changed during evolution.

Grylloblattidae live on the margins of glaciers, around lakes and marshes, on snow and ice surfaces, in ice caves, under rotten wood and debris in woodlands, and in caves. They are omnivorous and feed on other insects and occasionally on plants, fungi, and detritus (Pritchard & Scholefield, 1978). Grylloblattidae are nocturnal and are occasionally active during the day on the snow surface or on the ground. Living in groups is rare, and cannibalism occurs. Mated females usually devour their mates (Wang, 1999). All extant species are wingless, which explains their low mobility and dispersal capacity (Grimaldi & Engel, 2005; Marshall & Lytle, 2015). Grylloblattidae are adapted to low temperatures, the optimum living temperature being 0°C or slightly above, and the mortality rate increases significantly above 16°C.

The distribution of Grylloblattidae spans from 33°N to 60°N in the western part of the Rocky Mountains in North America (USA and Canada), northeastern Asia (the Changbai Mountains of China, North Korea, South Korea, Japan, and the southern coastal region of Russian Far East), and western Siberia (from the Altai to the Sayan Mountains). Studies have shown regional distribution at the genus level (Schoville, 2014; Schoville & Graening, 2013). For example, the genus *Grylloblatta* is distributed in northwestern North America, while the other five genera are distributed in Japan, the Korean Peninsula, China, the Pacific coast of Russia, the Altai Mountains, and the Sayan Mountains. Only two species, *Grylloblatta campodeiformis* Walker and *Galloisiana nipponensis* Caudell & King, are known to be widely distributed in the Rocky Mountains of North America and in Japan, respectively; all other species are restricted to the area of the type specimen with typical punctate distributions. Grylloblattidae are mostly distributed in low‐temperature high‐altitude areas (1500–3000 m) (Kamp, 1963) and in the cryosphere of low‐altitude areas (300–1000 m) (Kamp, 1979). Some species live in special habitats at lower altitudes (5–300 m) (Pravdin & Starozhenko, 1977; Schoville et al., 2013; Schoville & Roderick, 2010). For example, some species of *Grylloblatta*, *Namkungia*, and *Galloisiana* live in dense forests, and some are troglobites (Bai et al., 2010; Misof et al., 2014; Schoville et al., 2019).

Grylloblattidae are extremely rare in China. To date, only two specimens have been captured (Bai et al., 2010). In 1987, Mr. Shu‐Yong Wang collected the first specimen of a grylloblattid in the Changbai Mountains (Jilin Province, China), and this specimen was named *Galloisiana sinensis* Wang. The author provided a detailed morphological description of the specimen and showed that despite many similarities with *G. nipponensis* Caudell & King, there were many differential features such as a concave shape in the middle of the hind margin of the pronotum, fewer antennal segments, anterior legs with slender femora, more and denser setae on the inner margin beneath, and a different shape of the epiproct. The author also concluded that the species differed from *Grylloblattina kurentzovi* Pravdin & Storzhenko in that the middle of the hind margin of the pronotum protruded posteriorly in a horn shape; the species differed from the two species *Namkungia biryongensis* Namkung and *Galloisiana kosuensis* Namkung collected from caves in North Korea in that the two species had degenerated compound eyes (Kim & Lee, 2007; Wang, 1987). In 2009, Mr Ke‐qing Song collected the second grylloblattid at Lake Akekule (Lake Bai) in Kanas (Xinjiang, China), and the specimen was named *Grylloblattella cheni* Bai, Wang & Yang. This species was assigned to *Grylloblattella*, and the other two species of the genus are *Grylloblattella pravdini* Storozhenko & Oliger distributed in the Altai Mountains in western Siberia and *Grylloblattella sayanensis* Storozhenko in the Sayan Mountains in central Siberia (Wang, 1999). In 2010, a team led by Bai Ming in the Institute of Zoology, Chinese Academy of Sciences, conducted a systematic study of the second new species of Grylloblattidae discovered in China (*G. cheni* Bai, Wang & Yang). In addition to providing a distribution map and a key to the species of *Grylloblattella*, the authors investigated the thorax, especially the pronotum, of extant and extinct Grylloblattodea using geometric morphometric analysis. The results showed that the high diversity of extinct Grylloblattidae may reflect their diverse habitats and niches, and the diversity of modern Grylloblattidae could be explained by synapomorphy or convergent evolution. The authors also showed that most fossil Grylloblattidae had a clearly longer meso‐ and metathorax than prothorax, whereas modern Grylloblattidae have a shorter metathorax than prothorax, a phenotype related to the loss of wings and with the associated muscle reduction and changes in the thoracic skeleton system. Finally, the authors described some possible threats to the survival of Grylloblattidae and provided some suggestions for their conservation (Bai et al., 2010).

In summary, there are few studies on Grylloblattidae; their geographical distribution and phylogenetic status are significantly understudied, and their life history and biological characteristics are poorly understood. In particular, Grylloblattidae are extremely understudied in China, and type specimens are very scarce. In this study, we systematically investigated an extremely important new grylloblattid found near Ji'an City, Jilin Province, China. Morphological and molecular data were obtained to explore the phylogenetic status of Grylloblattidae and to confirm the phylogenetic relationships of the new genus with the other five genera and other insects. This paper adds a new genus to Grylloblattodea, provides important material for the determination of the phylogenetic status of Grylloblattodea, and enriches the morphological and molecular information for the study of Grylloblattodea evolution. This study is of significance for the origin and geographic dispersal of Grylloblattidae. In the future, industrial development, human activities, and global warming may threaten the reported and undiscovered Grylloblattidae, and this research will be relevant to the conservation of Grylloblattidae.

## 2 MATERIALS AND METHODS

### 2.1 Sampling

**FIGURE 1:**
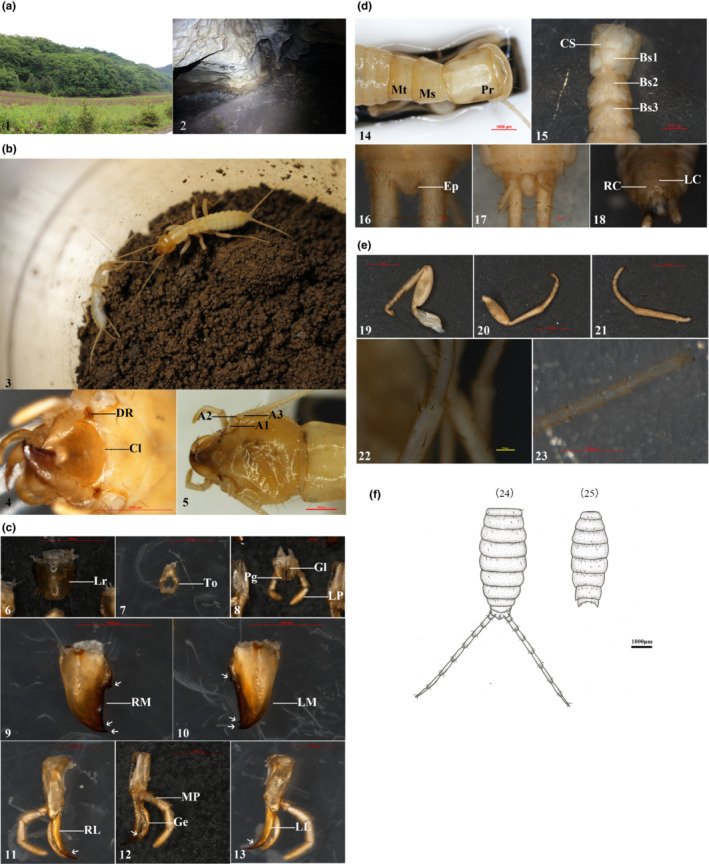
(a–f) Type localities, habitus, and structural characteristics of *Grylloprimevala jilina* sp. nov. (a) (1) habitat; (2) collection location. (b) (3) habitus, dorsal view; (4) anterior part of head, dorsal view; (5) head, dorsal view. A1, first antennomere; A2, second antennomere; A3, third antennomere; Cl, clypeus; DR, dark red funnel‐shaped structure. (c) (6) labrum, dorsal view; (7) tongue, dorsal view; (8) labium, dorsal view; (9) right mandible, dorsal view; (10) left mandible, dorsal view; (11) right maxillae, dorsal view; (12) right maxillae, ventral view; (13) left maxillae, dorsal view. Ge, galea; Gl, glossa; LL, left lacinia; LM, left mandible; LP, labial palp; Lr, labrum; MP, maxillary palp; Pg, paraglossa; RL, right lacinia; RM, right mandible; To, tongue. (d) (14) pronotum, mesonotum and metanotum, dorsal view; (15) cervical sclerites and basisternum, ventral view; (16) posterior part of abdominal, dorsal view; (17) posterior part of abdominal, ventral view; (18) posterior part of abdominal, ventral view; Bs1, first basisternite; Bs2, second basisternite; Bs3, 3rd basisternite; CS, cervical sclerite; Ep, epiproct; LC, left coxopodite; Ms, mesonotum; Mt, metanotum; Pr, pronotum; RC, right coxopodite. (e) (19) foreleg, dorsal view; (20) midleg, dorsal view; (21) hindleg, dorsal view; (22) spines, ventral view; (23) anterior part of tarsus, ventral view. (f) (24) abdominal tergites and cerci, dorsal view; (25) abdominal sternites, ventral view.

Specimens were found in good condition (vivid coloration, robust exoskeleton, and free movement) in a dark area of a natural cave in a primeval forest near Ji'an city, Jilin Province, China, located at 42°11′10.32″N, 123°43′22.75″E, 132 m above sea level (1 in Figure 1a). The location is a karst cave with no direct sunlight. At the bottom of the cave, there are small pools and mounds of earth and rocks, and stalactites on the roof and walls of the cave drip water year‐round. The cave temperature is 16.4°C, and the cave humidity is 85.2%. The collected rock piles were located 12 m from the entrance and 2.5 m below the surface (2 in Figure 1a). We collected specimens from May 19, 2020, to May 22, 2020, and June 19, 2021, to June 22, 2021. The new species has only been found in this natural cave in the primeval forest near Ji'an city, Jilin Province, China.

All specimens were collected by hand sampling. We used entomological tweezers and scoopulas (stainless steel spatulas used in chemistry) to protect the biological material.

Specimens were placed in insect boxes containing soil collected from the type locality. The insect box was temporarily placed in an incubator, and an ice pack was placed on either side of the insect box. The incubator was taken back to the laboratory.

### 2.2 Observations

The specimens were placed in a 40 cm × 30 cm × 30 cm ecological rearing tank. The soil collected from the type locality was placed in the tank, and a thermometer and hygrometer were also put into the tank. The ecological rearing tank was placed in an incubator with a consistent temperature of 16.4 ± 1°C and humidity of 85.2 ± 1%. The incubator was always kept in the dark.

The observation experiment lasted for 6 days and was divided into three stages. On the first and second days, we placed Diplura (as food) caught in the same cave with the specimens in the tank. From the third to the sixth day, Grylloblattidae were not fed. Infrared cameras (Sony Digital Handycam HDR‐CX760E) were used to record the conditions in the ecological rearing tank during the three periods of 07:00–09:00, 13:00–15:00, and 19:00–21:00 every day. The focal length of the lens was adjusted when the camera was set up to keep the pictures clear and the entire tank within the camera's view. The video data were then numbered, named, and saved in the computer.

Two researchers were assigned to play the video at normal speed (50 frames per second) and observe the recorded behaviors of the specimens.

### 2.3 Morphological analysis

This report is based on the study of the adult male specimens, described here as representing a new species. The specimens were identified using species descriptions of Grylloblattidae in Storozhenko (1988). All specimens in the type series were preserved in 70% ethanol. One holotype and two paratypes were collected. Voucher specimens and a research collection were deposited at Jilin Provincial Key Laboratory of Animal Resource Conservation and Utilization (Northeast Normal University, Changchun, China). Measurements are in millimeters unless noted otherwise.

Physical measurements and images were acquired using software (NIS‐Element) mounted on a stereozoom microscope (Nikon‐SMZ800N). Images were saved as TIF files, and editing and plate layout were performed in Adobe Photoshop. We followed Nagashima (1982, 1991), Ono (1988), Uchifune and Machida (2005), and Wang (1999) for terminology on Grylloblattidae morphological structures. Abbreviations used are as follows: A1, first antennomere; A2, second antennomere; A3, third antennomere; Bs1, 1st basisternite; Bs2, 2nd basisternite; Bs3, 3rd basisternite; Cl, clypeus; CS, cervical sclerite; DR, dark red funnel‐shaped structure; Ep, epiproct; Ge, galea; Gl, glossa; LC, left coxopodite; LL, left lacinia; LM, left mandible; LP, labial palp; Lr, labrum; MP, maxillary palp; Ms, mesonotum; Mt, metanotum; Pg, paraglossa; Pr, pronotum; RC, right coxopodite; RL, right lacinia; RM, right mandible; To, tongue.

### 2.4 Phylogenetic analysis

Genomic DNA was extracted from the legs of the two paratypes by the phenol chloroform method for subsequent genetic analysis. Six conserved genes (18 s rDNA, 28 s rDNA, Histone 3, 12 s rDNA, 16 s rDNA, and Cytochrome Oxidase II) were amplified by using Green Mix (TSE101; Tsingke, Beijing, China) (Skerratt et al., 2002; Svenson & Whiting, 2004; Whiting, 2002). Polymerase chain reaction (PCR) products were subjected to electrophoresis on 1% agarose gels, purified using a SanPrep Column DNA Gel Extraction Kit (B518131; Sangon Bio, Shanghai, China), linked to a plasmid using the *pEASY*®‐Blunt Zero Cloning Kit (CB501‐01, Transgen Bio, Beijing, China) and then transferred into *Trans‐T1* Phage Resistant Chemically Competent Cells (CD501‐02; Transgen Bio, Beijing, China). The monoclonal colonies were selected by overnight culture at 37°C followed by sequencing and splicing with general primers M13 (Sangon Sequencing) (Table 1). The sequences of other Grylloblattidae samples used to construct the phylogenetic tree were obtained from the NCBI database. After sequence alignment with PhyloSuite (Zhang et al., 2020), the best nucleotide substitution model was calculated by ModelFinder, and the maximum likelihood tree was constructed. The constructed evolutionary tree was imaged by FigTree (version 1.4.4).

**TABLE 1:** Primer sequences of the six conserved genes

Gene	Primer	Sequence	GenBank Accession number	Reference
18S	18S 1.2F	TGCTTGTCTCAAAGATTAAGC	OP942362	Whiting (2002)
	18S 9R	GATCCTTCCGCAGGTTCACCTAC		
28S	28S rD1.2a	CCCSSGTAATTTAAGCATATTA	OP942364	Whiting (2002)
	28S rD7b1	GACTTCCCTTACCTACAT		
H3	HexAF	ATGGCTCGTACCAAGCAGACGGC	OP924016	Svenson and Whiting (2004)
	HexAR	ATATCCTTGGGCATGATGGTGAC		
12S	SR‐N‐14199	TACTATGTTACGACTTAT	OP942363	Skerratt et al. (2002)
	SR‐N‐14594	AAACTAGGATTAGATACCC		
16S	16Sa	CGCCTGTTTATCAAAAACAT	OP942365	Svenson and Whiting (2004)
	16Sb	CTCCGGTTTGAACTCAGATCA		
CO II	COII Flue	TCTAATATGGCAGATTAGTGC	OP924017	Svenson and Whiting (2004)
	COII R‐lys	GAGACCAGTACTTGCTTTCAGTCATC		

## 3 RESULTS

**Family Grylloblattidae Walker, 1914.**

**Genus *Grylloprimevala* Zhou & Ren, gen. nov.**

### 3.1 Type species

*Grylloprimevala jilina* Zhou & Ren, sp. nov., here designated.

### 3.2 Diagnosis of the new genus

Posterior margin of pronotum slightly concave, without poorly sclerotized zones. Cervical sclerite with six setae on internal margin and nine setae on external margin. Lacinia with one tooth. No pulvilli on the tarsal segments. Male epiproct symmetrical. Median projection on posterior margin of male epiproct very short, acutely pointed, triangular, and with a median depression.

### 3.3 Composition

The genus is monotypic.

### 3.4 Etymology

The new genus was named after the primitive features.

***Grylloprimevala jilina* Zhou & Ren, sp. nov.**
LSID: urn:lsid:zoobank.org:pub:E4C45C1D‐CBB0‐4298‐AC87‐B09FD5FB7826.

### 3.5 Type locality

Located in a dark area of a natural cave in a primeval forest near Ji'an city, Jilin Province, China.

### 3.6 Type material

Holotype, 1♂, China, Jilin Province, Ji ‘an, dark zone of a natural cave in a primeval forest, 42°11′10.32″N, 123°43′22.75″E, 132 m above sea level, sunny, May 19, 2022 (Jilin Provincial Key Laboratory of Animal Resource Conservation and Utilization), NENU2020M0001, Gatherer: Lin Zhou; Collector: Bingzhong Ren. Paratypes, 2♂, China, Jilin Province, Ji ‘an, dark zone of a natural cave in a primeval forest, 42°11′10.32″N, 123°43′22.75″E, 132 m above sea level, sunny, June 19, 2021 (Jilin Provincial Key Laboratory of Animal Resource Conservation and Utilization), NENU2021M0002, NENU2021M0003, Gatherer: Lin Zhou; Collector: Bingzhong Ren.

### 3.7 Diagnosis of the new species

Antennae composed of 39 antennomeres; length of first antennomere about 2.43 times the length of the second. Compound eyes severely degenerated and replaced by dark red funnel‐shaped structures. No ocellus. Lacinia with one tooth. Posterior margin of pronotum slightly concave, without poorly sclerotized zones. Cervical sclerite with six setae on internal margin and nine setae on external margin. No pulvilli on the tarsal segments. Male epiproct symmetrical. Median projection on posterior margin of male epiproct very short, acutely pointed, triangular, and with a median depression. Cerci composed of nine cercomeres.

### 3.8 Description of the holotype male

The total body length is 14.38 mm (the distance from the insertion of the cerci to the clypeus suture). The body is slender and buff yellow; the surface of the body is smooth, is densely covered with fine, short, light brown hairs, and sparsely covered with dark brown setae (3 in Figure 1b).

Head: prognathous head. The cranium (length = 2.89 mm, width = 2.23 mm) is about 1.3 times as wide as the pronotum, with seven setae on each lateral and posterior margin, four setae on the center and uniformly distributed on both sides of the epicranial suture, and four setae around the antennal socket (5 in Figure 1b). The epicranial suture is Y‐shaped, distinct, and connected to the circumantennal suture, with two parietal sutures extending from the occipital foramen to the vertex (5 in Figure 1b). The clypeus is wider than it is long (length = 0.21 mm, width = 0.51 mm), is projected on the cranium anterior middle section, and is divided into an anterior and posterior clypeus. The anterior clypeus is membranous, and the posterior clypeus is sclerotized (4 in Figure 1b). The compound eyes are severely degenerated, with only a dark red funnel‐shaped structure without an ocellus remaining (4 in Figure 1b). The antennae (length = 8.91 mm) are filiform, slender, and approximately equal to the sum of the lengths of the head, thorax, first abdominal tergites, and second abdominal tergites. The antennae are composed of 39 antennomeres. The first antennomere (0.64 mm) is the largest; and its length is twice its width. The second antennomere (0.21 mm) is shorter, one‐third the length of the first. The third antennomere (0.51 mm) is longer than the second. The 4th–7th antennomeres are shorter, and the length of each segment is nearly equal to its width. The 8th–39th antennomeres gradually become elongated. There are sparse hairs at the bases of the first and second antennomeres and dense hairs on the remaining antennomeres (5 in Figure 1b).

Chewing mouthparts: The labrum is developed. The length is equal to the width; the base is broad and is attached to the clypeus; and the surface is well‐sclerotized and covered with dark brown setae (6 in Figure 1c). The tongue is shaped like an oval bowl and membranous; the base is slightly narrower than the distal; and the surface is slightly sclerotized and glabrous (7 in Figure 1c). Mandibles are developed (length = 1.14 mm, width = 0.69 mm), symmetrical, and highly sclerotized. There are two teeth in the distal part and one tooth on the inner margin, and there is no grinding area (9–10 in Figure 1c). The maxillae are larger (length = 2.09 mm, width = 0.61 mm). The distal part extends past the top of the mandibles. They are composed of the cardo, stipes, lacinia, galea, and maxillary palpus. The structure belongs to Orthoptera (11–13 in Figure 1c). The galea is sickle‐shaped (length = 0.88 mm, width = 0.11 mm). The distal part is strongly curved and weakly sclerotized and has a semicircular shape with serrated edges on the inner top and scattered dark brown setae on the inner margin (12 in Figure 1c). The lacinia is similar in shape to the galea (length 0.98 mm, width 0.21 mm). It is curved, dark in color, and strongly sclerotized. There is one preapical tooth, the base does not contain teeth, there is one row composed of four dark brown setae between the base and apical teeth (11 and 13 in Figure 1c). The maxillary palp has five palpomeres and is 1.98 mm (0.14 mm + 0.16 mm + 0.48 mm + 0.48 mm + 0.72 mm) long. The first palpomere is the shortest, and the second is one‐third the length of the third. The length of the third is the same as that of the fourth; and the fifth is the longest (1.5 times longer than the fourth) and is densely covered with fine, short, light brown hairs (12 in Figure 1c). The labium has a pair of labial palps, a pair of paraglossae and a pair of smaller glossae (8 in Figure 1c). There are three segments in the labial palps, with a total length of 0.97 mm (0.23 mm + 0.25 mm + 0.49 mm). The first and second segments contain a few dark brown setae, and the third is covered with many fine, short, light brown hairs (8 in Figure 1c). There is a row of dark brown setae on the outer margin of the paraglossae and glossae without setae (8 in Figure 1c).

The pronotum is 1.13 times as long as it is wide (length = 2.23 mm, width = 1.97 mm) and is quadrilateral. The anterior part of the pronotum has a deep transverse sulcus, is elongated anteriorly, lacks poorly sclerotized zones, and has some dark brown setae on its anterior margin and scattered dark brown setae on its dorsum. The longitudinal suture is continuous and long (14 in Figure 1d). The mesonotum is 1.09 times as long as it is wide (length = 1.32 mm, width = 1.21 mm), has scattered dark brown setae on its dorsum, and lacks a longitudinal suture (14 in Figure 1d). The metanotum 1.95 times as wide as it is long (length = 0.93 mm, width = 1.81 mm), has scattered dark brown setae on the posterior margin, and lacks a longitudinal suture (14 in Figure 1d). The posterior margin of the pronotum is slightly concave, and the mesonotum and metanotum are broadly rounded and clearly concave in the posterior part.

The cervical sclerites is about twice as long as it is wide (length = 1.12 mm, width = 0.56 mm), is broadly triangular and elongated anteriorly, and has six dark brown setae on the inner margin and nine dark brown setae on lateral margin (15 in Figure 1d). The basisternum of the prothorax is 1.38 times as wide as it is long (length = 0.74 mm, width = 1.02 mm), is triangular, and has a long median suture and four dark brown setae on the anterior part (15 in Figure 1d). The basisternum of the mesothorax is 1.7 times as wide as it is long (length = 0.56 mm, width = 0.96 mm) and has a median suture originating posteriorly and many scattered setae (15 in Figure 1d). The basisternum of the metathorax is about 2.67 times as wide as it is long (length = 0.36 mm, width = 0.96 mm) and has a short median suture originating posteriorly and many scattered setae (15 in Figure 1d).

There are three pairs of legs, including strong forelegs (7.81 mm), and elongated midlegs (7.26 mm) and hindlegs (8.27 mm) (19–21 in Figure 1e). The coxa is large and strong with a distinct rib. The femur of the foreleg is thick, is about 2.8 times as long as it is wide (length = 2.71 mm, width = 0.97 mm), and has a smooth dorsum and scattered dark brown setae. The length of the foreleg femur is 1.15 times that of the midleg femur (length = 2.36 mm, width = 0.83 mm), and the length of the hindleg femur (length = 2.69 mm, width = 1.06 mm) is 1.14 times that of the midleg femur. The tibia is slightly shorter than the femur, and there are two rows of dark brown setae neatly distributed on the inner margin of the ventral surface. The apical part of the tibia contains two large spines. The medial spines are elongated, and the lateral spines are robust (22 in Figure 1e). The tarsus has five segments. The first to fourth ventral front segments have a pair of dark brown setae on both sides and no euplantulae. The fifth segment has no euplantulae on the ventral surface of the apical part, one pair of strong tarsal claws, and no pulvilli or arolium (23 in Figure 1e).

The abdominal tergites (24 in Figure 1f) contain 10 segments and are slightly convex in the middle of the posterior margin. There are seven setae on the dorsal part of the 1st tergite, six setae on the dorsal part of the second and third tergites, four on the fourth tergite, five on the fifth tergite, three on the sixth and eighth tergites, and one on the seventh tergite. The first tergite is about 4.69 times as wide as it is long (length = 0.46 mm, width = 2.16 mm), the second is 4.21 times as wide as it is long (length = 0.56 mm, width = 2.36 mm), the third is 4.21 times as wide as it is long (length = 0.58 mm, width = 2.44 mm), the fourth is 3.81 times as wide as it is long (length = 0.62 mm, width = 2.38 mm), the fifth is 4.11 times as wide as it is long (length = 0.54 mm, width = 2.22 mm), the sixth is 4.04 times as wide as it is long (length = 0.48 mm, width = 1.94 mm), the seventh is 3.98 times as wide as it is long (length = 0.48 mm, width = 1.91 mm), and the eighth is 5.21 times as wide as it is long (length = 0.28 mm, width = 1.46 mm).

The abdominal sternites (25 in Figure 1f) are slightly convex in the middle of the posterior margin, and there are scattered dark brown setae on the anterior part and posterior part. The first sternite is about 3.31 times wide as it is long (length = 0.46 mm, width = 1.52 mm), the second is 3.00 times as wide as it is long (length = 0.56 mm, width = 1.68 mm), the third is 3.03 times as wide as it is long (length = 0.58 mm, width = 1.76 mm), the fourth is 2.81 times as wide as it is long (length = 0.62 mm, width = 1.74 mm), the fifth is 3.06 times as wide as it is long (length = 0.53 mm, width = 1.62 mm), the sixth is 3.04 times as wide as it is long (length = 0.48 mm, width = 1.46 mm), the seventh is 2.58 times as wide as it is long (length = 0.48 mm, width = 1.24 mm), the eighth is 2.33 times as wide as it is long (length = 0.48 mm, width = 1.12 mm), and the ninth is 2.97 times as wide as it is long (length = 0.32 mm, width = 0.95 mm).

The 10th abdominal sternite (length 0.32 = mm, width = 0.75 mm) has a pair of slightly curved symmetrical apical processes, a pair of oval paraprocts, and an epiproct (17 in Figure 1d). The epiproct of the male is symmetrical with an acutely pointed triangular median projection on the posterior margin and a median depression (16 in Figure 1d).

The cercomeres (3.11 mm) (24 in Figure 1f) have nine segments and are filiform. The lengths of the first and second are equal, length gradually increases from the third to ninth, and the last section is elongated. The cerci is hollow; there are a small number of dark brown setae at the end of each segment, and there are long, thick, dark brown setae at the end of the last segmentare.

The coxopodites are asymmetrical. The right coxopodite is longer than it is wide and sclerotized (length = 0.48 mm, width = 0.32 mm), and the inner side and the basal segment are narrow. The left coxopodite is wider than it is long (length = 0.29 mm, width = 0.36 mm), is membranous, has a strong curved scoop‐like shape. The subgenital plate is wider than it its long, and the distal edge is hardened (18 in Figure 1d).

### 3.9 Behavior

According to the observations, *G. jilina* sp. nov. often stop to clean their antennae, legs, and cerci with their mouthparts when crawling in order to keep their surface sensors clean and sensitive. As individuals crawl, their antennae vibrate in the air and touch the soil and obstacles to discern direction and to search for food. When two individuals of the same species meet, they sometimes gather in one place and constantly touch each other's antennae or body for a period of time to complete recognition and information communication. Sometimes when the antennae of two individuals come into contact, both insects quickly flee. This constant contact and rapid escape occur without regularity. After placing *Anisuracampa ywangana* Sendra & Komerički (Diplura) collected in the same cave as the individual in the rearing tank, the individual was found to prey on the diplurans. When there were no other insects in the feeding tank, the individual used insect residues and other organic matter in the soil as food. After the insect residues and other organic matter that could be found in the soil and the diplurans were eaten, individuals began to prey on each other, but they did not prey on each other every time they met, and their walking power decreased significantly after satiation. The insects were usually stationary in one place, crawled a short distance after being disturbed, and then returned to a stationary state.

### 3.10 Phylogenetic relationships

**FIGURE 2:**
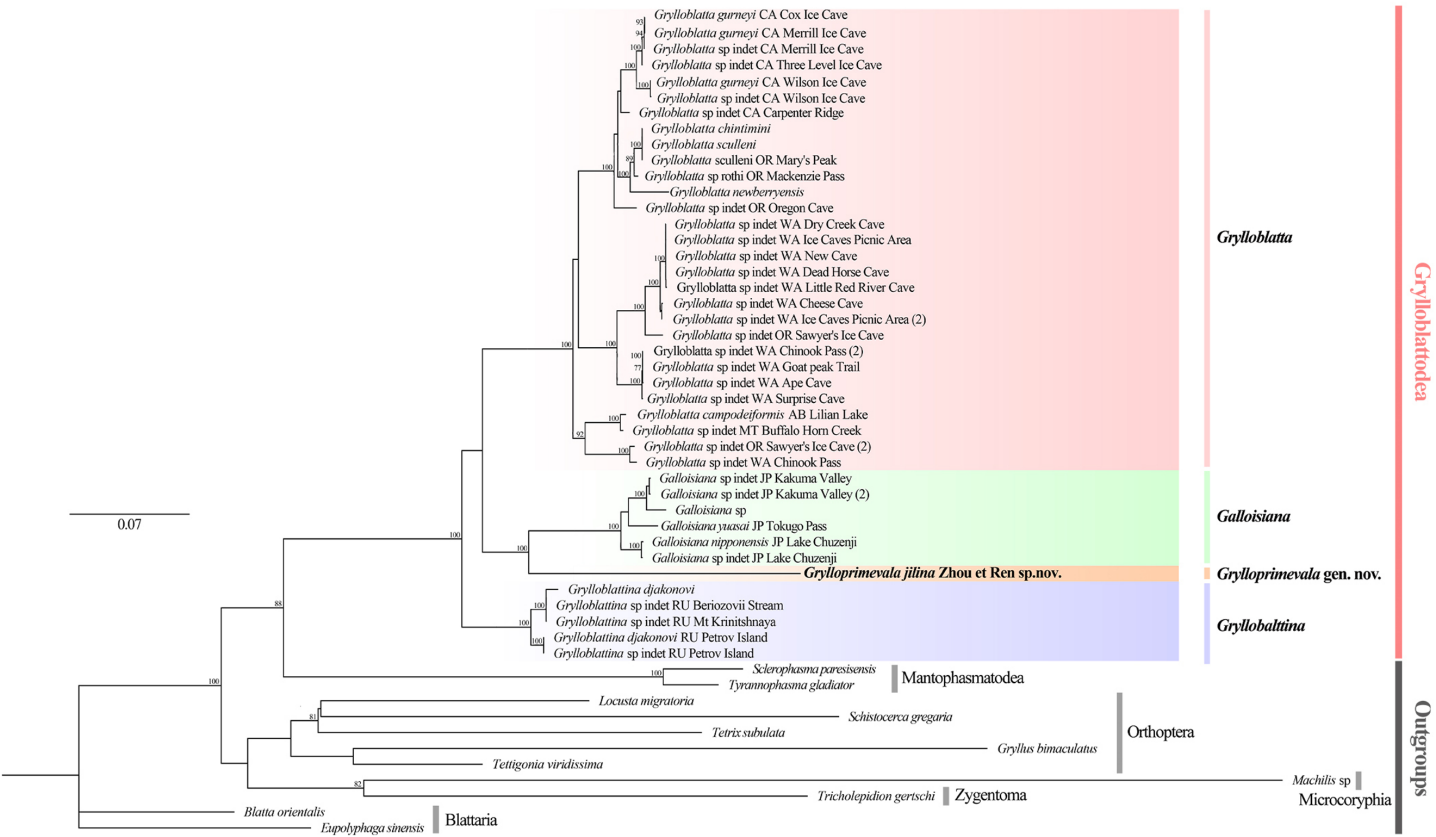
Systematic relationships of different genera among the Grylloblattidae. Build the maximum likelihood tree, set the bootstrap replications to 1000.

A maximum likelihood tree was constructed based on the joint dataset of 18 s, 28 s, H3, 12 s, 16 s, and COII genes (Figure 2). Species of Mantophasmatodea, Orthoptera, Zygentoma, Microcoryphia, and Blattodea were selected as outgroups. The topological structure of the evolutionary tree was consistent with the tree constructed by Jarvis and Whiting (Table S1) (Jarvis & Whiting, 2006). In the molecular phylogenetic tree, all species of Grylloblattidae were grouped into a large clade, supporting the monophyly of Grylloblattidae. In Grylloblattidae, the genus *Grylloblattina* first differentiated and formed sister branches with other groups of Grylloblattidae. *Grylloprimevala* gen. nov. and *Galloisiana* formed a sister group first, and further clustered as one clade with G*rylloblatta*.

### 3.11 Etymology

The specific epithet was named after the habitat and collection site of the new species from Jilin Province, China.

## 4 DISCUSSION

### 4.1 Behavior

Our observations on the Grylloblattid individuals revealed that *G. jilina* sp. nov. used their mouthparts to clean the antennae, cerci, and legs. Insects communicated by touching antenna. Two individuals occasionally gathered in one place and gently touched each other's body for a period of time, and at times they immediately fled from each other after antenna contact. These observations indicated independence from the eyes and dependence on the function of body sensors, and a willingness to spend a significant amount of time keeping the body sensors clean and sensitive. Cannibalism was observed, but not after every contact between individuals. The mobility of individuals was significantly reduced after satiation. These behaviors exhibited by individuals under food scarcity were derived from the evolution of troglobites to ensure nutritional intake and reduce energy consumption in order to survive in extreme environments. Taken together, these behaviors differed from those of most Grylloblattidae but resembled troglobites living in the dark zones of caves and thus are important for studying the survival and evolution of Grylloblattidae.

### 4.2 Comparisons

*Grylloprimevala* gen. nov. in this study is different from other known genera previously discovered.

Compared with *Grylloprimevala* gen. nov., *Grylloblattella*, *Namkungia*, *Grylloblattina*, and *Galloisiana* have one more tooth in lacinia. *Grylloblattella*, *Namkungia*, and *Galloisiana* have less setae on the internal margin of cervical sclerite. Only *Grylloprimevala* gen. nov. without pulvilli on the tarsal segments. Furthermore, the posterior margin of supra‐anal plate is symmetrical in *Grylloprimevala* gen. nov., *Grylloblattella*, *Namkungia* and *Grylloblattina*, and asymmetrical in *Grylloblatta* and *Galloisiana*.

In conclusion, based on the morphological data, it can be seen that *Grylloprimevala* gen. nov. is significantly different from the other five existing genera. This shows clear signs of adaptation to the dark cave environment, and the species displayed unique cave behavior characteristics. The directional selection of cave environments makes its distribution appear sporadic and spot‐like. Combined with the genetic data, the *Crylloprimevala* gen. nov. is a unique evolutionary lineage that is significantly different from the other five known genera of Grylloblattidae. Therefore, it is necessary to establish a new genus and species to better classify Grylloblattidae.

### 4.3 Ecological environment of distribution area

Almost all Grylloblattidae live in an environment characterized by low temperature throughout the year, snow coverage, and high humidity (Kamp, 1963, 1979; Vrsansky et al., 2001). As temperatures become near zero, Grylloblattidae are active during the night, and they usually climb to snowfields after sunset to forage and hide in dark areas such as rock crevices during the day (Mann et al., 1980; Schoville, 2010). The optimal living temperature of Grylloblattidae is between 0 and 1°C, and mortality increases significantly above 16°C; they rarely survive at temperatures below −6.2°C or above 20.5°C (Edwards, 1982; Henson, 1957). Although little is known about the mechanism of action of temperature on their activities, a study of *G. jilina* sp. nov. in the laboratory showed that they preferred 16.4 ± 1°C, survived between 0 and 23°C, and died at temperatures >25°C. In alpine habitats, Grylloblattidae are mostly found on rocky grounds formed by weathering and in basins covered with snow (Schoville, 2010). At low altitudes, Grylloblattidae live in caves/lava tubes, canyons, and rocky river banks (Edwards, 1982). The new species *G. jilina* sp. nov. reported in this paper was found in a natural cave in a primary forest near Ji'an City, a habitat that is cold throughout the year, humid, dark, and rarely visited by humans; the location could be regarded as an isolated island habitat with little material and energy exchange with the surrounding environment. *G. jilina* sp. nov. had poor dispersal ability. We investigated the above‐ground environment and similar caves in the area and did not find other individuals of the species. Thus, this spatial pattern suggests that *G. jilina* sp. nov. is a species restricted to the cave. The species is characterized by extremely low population abundance, extremely poor dispersal and migration ability, an extremely narrow distribution, a clear preference for the specific environment, and extremely low stress resistance. Thus, the *G. jilina* sp. nov. population is fragile, nonresilient, and extremely rare.

### 4.4 Phylogenetic relationships

Recent Grylloblattodea is a small order of insects comprising one family and six genera (including *Grylloprimevala* gen. nov.). Individuals in this order are rare, and samples are difficult to obtain. As such, most studies are based on morphological data and lack relevant molecular tags. According to the molecular data of three genera obtained from previous studies, the conservative sequence of *G. jilina* sp. nov. was obtained using universal primers. The results of the combined phylogenetic tree construction showed that the species of the four genera were clustered into one clade. The genus *Grylloblattina* was the first to differentiate, and the genera *Grylloprimevala* gen. nov. and *Galloisiana* were clustered into a large branch, indicating that their genetic relationship may be closer. Three species of Grylloblattidae (including the new species described in this study) are known in China, and they belong to three different genera. As the first known and named one, *G. sinensis* Wang was found in the Changbai Mountains. *G. sinensis* Wang living outside the cave are dark and have developed compound eyes. *G. jilina* sp. nov. also lives in the Changbai Mountains. Both species lack wings and have weak mobility and a similar geographical distribution. They constitute sister branches in the evolutionary tree, indicating a close relationship. However, unlike species in the genus *Galloisiana*, the *G. jilina* sp. nov. lack compound eyes, which have been completely degraded; this may be related to their adaptations for living in a cave environment without direct sunlight. Combined with the unique morphological characteristics and molecular evolution analysis, *G. jilina* sp. nov. is regarded as the new genus *Grylloprimevala* gen. nov. The results of the molecular evolutional tree analysis indicate that Grylloblattodea and Mantophasmatodea constitute sister branches and suggest that they may be closely related sister groups. However, more morphological characteristics and molecular data are needed to verify this hypothesis.

### 4.5 Conservation

Because the microhabitat of *G. jilina* sp. nov. is highly specialized and the species is characterized by scarcity, low abundance, impaired migration, and limited geographical distribution, several recent conservation assessments have concluded that *G. jilina* sp. nov. can be considered as an endangered species and that its survival is threatened. Climate change (e.g., global warming, dramatic changes in local precipitation, changes in snow cover, and sustained increase in mean annual temperature), anthropogenic damage, and landscape change will directly affect the habitat of *G. jilina* sp. nov. and lead to its extinction.

Currently, several important measures should be taken to update and improve the conservation and assessment of *G. jilina* sp. nov. First, an accurate distribution pattern of the new species should be determined before investigation of syntopic occurrence (occurrence of different species at the same locality). Second, long‐term investigations for conservation should be conducted at the type specimen distribution site, including the cave where the specimen was found, the ground environment outside the cave, and all caves within a radius of 50 km. The conservation status we assigned to this novel species provides updated data for the NatureServe database and the IUCN Red List.

### 4.6 Future research

We currently have sufficient information to determine the activity location, to collect samples, and to monitor the population of *G. jilina* sp. nov. The challenges in the coming years involve addressing several prominent issues in the systematic study of *G. jilina* sp. nov. These include: (1) to thoroughly explore the mechanisms of compensatory evolution of sensors of *G. jilina* sp. nov. in the dark cave in response to the degradation of eyes; (2) to obtain more morphological and molecular data with which to clarify the status of this species in Grylloblattodea; (3) to investigate the phylogenetic status of Grylloblattodea in Insecta from a genomic point of view; (4) to correlate the origin of Grylloblattodea with the melting of glaciers and the formation of the Changbai Mountain refuge; (5) to search for other individuals of *G. jilina* sp. nov. in the caves surrounding the type specimen distribution site; and (6) to reasonably assess the endangerment of *G. jilina* sp. nov. with government authorities and to establish a comprehensive conservation strategy. The resolution of these issues will provide a robust systematic framework for exploring the evolution and ecology of Grylloblattodea species, especially *G. jilina* sp. nov.

## 5 CONCLUSION

In this study, we describe a very rare new species of Grylloblattidae that adds to the extant taxa of Grylloblattodea. Our results provide important data for subsequent construction of a morphology‐based phylogenetic tree of Grylloblattodea and the study of the relationships between Grylloblattodea species. Tissue samples were collected to obtain molecular data that provide important information for determining the phylogenetic status of Grylloblattodea and important material for the study of Grylloblattodea evolution. Our findings are relevant to research on the origin, distribution, and dispersal of Grylloblattodea. This study provides basic data for the conservation of Grylloblattodea diversity and habitat.

## AUTHOR CONTRIBUTIONS

**Lin Zhou:** Conceptualization (equal); data curation (equal); formal analysis (equal); investigation (equal); methodology (equal); software (equal); visualization (equal); writing – original draft (equal). **Qi Chen:** Conceptualization (equal); data curation (equal); formal analysis (equal); investigation (equal); methodology (equal); writing – review and editing (equal). **Haoqin Ke:** Investigation (equal); software (equal); visualization (equal). **Zizhuo Wang:** Software (equal); visualization (equal). **Jie Peng:** Investigation (equal). **Donghui Wu:** Investigation (equal). **Ying Liu:** Funding acquisition (equal); project administration (equal); resources (equal); supervision (equal). **Jiang Feng:** Resources (equal); supervision (equal). **Bingzhong Ren:** Conceptualization (equal); funding acquisition (equal); investigation (equal); project administration (equal); resources (equal); supervision (equal).

## CONFLICT OF INTEREST

The authors have no relevant financial or nonfinancial interest to disclose.

## Supporting information


Table S1

## Data Availability

The data underlying this article are available in the article and in its supporting information. DNA sequences and related data are publicly available on the National Center for Biotechnology public databases (https://www.ncbi.nlm.nih.gov/). The data associated with each of the specimens examined are included in the text, in the appropriate sections. Accession number for sequences downloaded from public databases is included in Table S1. Accession number for sequences those generated for this project is included in Table 1.
