# Supplementary material for: Descriptions of a new genus and a new species, Grylloprimevala jilina (Grylloblattidae) from China
Source: Ecol Evol. 2023 Jan 19;13(1):e9750. doi: 10.1002/ece3.9750 (PMC9852939; doi:10.1002/ece3.9750)
Supplement: Supplementary file 1 — Table S1 [file ECE3-13-e9750-s001.docx]

**Supporting information**

**Table S1** Taxa included in this analysis and GenBank accession numbers (absent sequence data listed as NA).

| Order | Family | Genus | Species | 18S rDNA | 28S rDNA | Histone 3 | 12S rDNA | 16S rDNA | Cytochrome Oxidase II |
| --- | --- | --- | --- | --- | --- | --- | --- | --- | --- |
| Grylloblattodea | Grylloblattidae | *Grylloblatta* | *Grylloblatta campodeiformis* AB Lilian Lake | DQ457300 | NA | NA | DQ457228 | DQ457263 | NA |
| Grylloblattodea | Grylloblattidae | *Grylloblatta* | *Grylloblatta* sp. indet. MT Buffalo Horn Creek | DQ457299 | DQ457336 | DQ457398 | DQ457227 | DQ457262 | DQ457367 |
| Grylloblattodea | Grylloblattidae | *Grylloblatta* | *Grylloblatta* sp. indet. WA Goat peak Trail | DQ457292 | DQ457329 | NA | DQ457220 | DQ457255 | DQ457362 |
| Grylloblattodea | Grylloblattidae | *Grylloblatta* | *Grylloblatta* sp. indet. WA Chinook Pass | DQ457274 | DQ457311 | DQ457376 | DQ457203 | DQ457237 | DQ457345 |
| Grylloblattodea | Grylloblattidae | *Grylloblatta* | *Grylloblatta* sp. indet. WA Chinook Pass（2） | DQ457275 | DQ457312 | DQ457377 | DQ457204 | DQ457238 | DQ457346 |
| Grylloblattodea | Grylloblattidae | *Grylloblatta* | *Grylloblatta* sp. indet. WA Dead Horse Cave | DQ457293 | DQ457330 | DQ457394 | DQ457221 | DQ457256 | DQ457363 |
| Grylloblattodea | Grylloblattidae | *Grylloblatta* | *Grylloblatta* sp. indet. WA New Cave | DQ457295 | DQ457332 | DQ457395 | DQ457223 | DQ457258 | DQ457365 |
| Grylloblattodea | Grylloblattidae | *Grylloblatta* | *Grylloblatta* sp. indet. WA Cheese Cave | DQ457289 | DQ457326 | DQ457391 | DQ457217 | DQ457252 | DQ457359 |
| Grylloblattodea | Grylloblattidae | *Grylloblatta* | *Grylloblatta* sp. indet. WA Ice Caves Picnic Area | DQ457272 | DQ457309 | DQ457374 | DQ457201 | DQ457235 | DQ457343 |
| Grylloblattodea | Grylloblattidae | *Grylloblatta* | *Grylloblatta* sp. indet. WA Ice Caves Picnic Area（2） | DQ457273 | DQ457310 | DQ457375 | DQ457202 | DQ457236 | DQ457344 |
| Grylloblattodea | Grylloblattidae | *Grylloblatta* | *Grylloblatta* sp. indet. WA Dry Creek Cave | DQ457291 | DQ457328 | DQ457393 | DQ457219 | DQ457254 | DQ457361 |
| Grylloblattodea | Grylloblattidae | *Grylloblatta* | *Grylloblatta* sp. indet. WA Surprise Cave | DQ457294 | DQ457331 | NA | DQ457222 | DQ457257 | DQ457364 |
| Grylloblattodea | Grylloblattidae | *Grylloblatta* | *Grylloblatta* sp. indet. WA Little Red River Cave | DQ457290 | DQ457327 | DQ457392 | DQ457218 | DQ457253 | DQ457360 |
| Grylloblattodea | Grylloblattidae | *Grylloblatta* | *Grylloblatta* sp. indet. WA Ape Cave | DQ457297 | DQ457334 | DQ457396 | DQ457225 | DQ457260 | NA |
| Grylloblattodea | Grylloblattidae | *Grylloblatta* | *Grylloblatta sculleni* OR Mary’s Peak | DQ457301 | NA | NA | DQ457229 | DQ457264 | DQ457368 |
| Grylloblattodea | Grylloblattidae | *Grylloblatta* | *Grylloblatta* sp. indet. OR Sawyer’s Ice Cave | DQ457270 | DQ457307 | DQ457372 | DQ457199 | DQ457234 | DQ457342 |
| Grylloblattodea | Grylloblattidae | *Grylloblatta* | *Grylloblatta* sp. indet. OR Sawyer’s Ice Cave（2） | DQ457271 | DQ457308 | DQ457373 | DQ457200 | NA | NA |
| Grylloblattodea | Grylloblattidae | *Grylloblatta* | *Grylloblatta* sp. *rothi.* OR Mackenzie Pass | DQ457269 | DQ457306 | DQ457371 | DQ457198 | DQ457233 | NA |
| Grylloblattodea | Grylloblattidae | *Grylloblatta* | *Grylloblatta* sp. indet. OR Oregon Cave | DQ457298 | DQ457335 | DQ457397 | DQ457226 | DQ457261 | DQ457366 |
| Grylloblattodea | Grylloblattidae | *Grylloblatta* | *Grylloblatta* sp. indet. CA Three Level Ice Cave | DQ457296 | DQ457333 | NA | DQ457224 | DQ457259 | NA |
| Grylloblattodea | Grylloblattidae | *Grylloblatta* | *Grylloblatta* sp. indet. CA Merrill Ice Cave | DQ457276 | DQ457313 | DQ457378 | DQ457205 | DQ457239 | DQ457347 |
| Grylloblattodea | Grylloblattidae | *Grylloblatta* | *Grylloblatta gurneyi* CA Merrill Ice Cave | DQ457277 | DQ457314 | DQ457379 | DQ457206 | DQ457240 | DQ457348 |
| Grylloblattodea | Grylloblattidae | *Grylloblatta* | *Grylloblatta gurneyi* CA Cox Ice Cave | DQ457278 | DQ457315 | DQ457380 | DQ457207 | DQ457241 | DQ457349 |
| Grylloblattodea | Grylloblattidae | *Grylloblatta* | *Grylloblatta gurneyi* CA Wilson Ice Cave | DQ457267 | DQ457304 | NA | DQ457196 | DQ457231 | DQ457339 |
| Grylloblattodea | Grylloblattidae | *Grylloblatta* | *Grylloblatta* sp. indet. CA Wilson Ice Cave | DQ457268 | DQ457305 | DQ457370 | DQ457197 | DQ457232 | DQ457340 |
| Grylloblattodea | Grylloblattidae | *Grylloblatta* | *Grylloblatta* sp. indet. CA Carpenter Ridge | DQ457288 | DQ457325 | DQ457390 | DQ457216 | DQ457251 | NA |
| Grylloblattodea | Grylloblattidae | *Galloisiana* | *Galloisiana* sp. | AY707341 AY707367 | AY707388 AY707408 AY707423 | AY707443 | NA | NA | NA |
| Grylloblattodea | Grylloblattidae | *Galloisiana* | *Galloisiana* sp. indet. JP Kakuma Valley | DQ457280 | DQ457317 | DQ457382 | DQ457209 | DQ457243 | DQ457351 |
| Grylloblattodea | Grylloblattidae | *Galloisiana* | *Galloisiana* sp. indet. JP Kakuma Valley（2） | DQ457281 | DQ457318 | DQ457383 | DQ457210 | DQ457244 | DQ457352 |
| Grylloblattodea | Grylloblattidae | *Galloisiana* | *Galloisiana nipponensis* JP Lake Chuzenji | DQ457282 | DQ457319 | DQ457384 | DQ457211 | DQ457245 | DQ457353 |
| Grylloblattodea | Grylloblattidae | *Galloisiana* | *Galloisiana* sp. indet. JP Lake Chuzenji | DQ457283 | DQ457320 | DQ457385 | NA | DQ457246 | DQ457354 |
| Grylloblattodea | Grylloblattidae | *Grylloblattina* | *Grylloblattina djakonovi* | AY707342  AY707368 | AY707389 AY707409 AY707424 | AY707444 | NA | NA | NA |
| Grylloblattodea | Grylloblattidae | *Grylloblattina* | *Grylloblattina* sp. indet. RU Petrov Island | DQ457285 | DQ457322 | DQ457387 | DQ457213 | DQ457248 | DQ457356 |
| Grylloblattodea | Grylloblattidae | *Grylloblattina* | *Grylloblattina* sp. indet. RU Beriozovii Stream | DQ457286 | DQ457323 | DQ457388 | DQ457214 | DQ457249 | DQ457357 |
| Grylloblattodea | Grylloblattidae | *Grylloblattina* | *Grylloblattina* sp. indet. RU Mt. Krinitshnaya | DQ457287 | DQ457324 | DQ457389 | DQ457215 | DQ457250 | DQ457358 |
| Grylloblattodea | Grylloblattidae | *Grylloblatta* | *Grylloblatta sculleni* | DQ457301 | KM853520 | NA | DQ457229 | DQ457264 | DQ457368 |
| Grylloblattodea | Grylloblattidae | *Grylloblatta* | *Grylloblatta chintimini* | NA | NA | NA | NA | NA | KF880960 |
| Grylloblattodea | Grylloblattidae | *Grylloblatta* | *Grylloblatta newberryensis* | NA | NA | NA | NA | NANA | KF880958 |
| Orthoptera | Tettigoniidea | *Tettigonia* | *Tettigonia viridissima* | KX429795 | KX429846 | KX429936 | Z97606 | Z97622 | KX429886 |
| Orthoptera | Gryllidae | *Gryllus* | *Gryllus bimaculatus* | AF514548 | KM508879 | KR903154 | KR903835 | JX269093 | KU705555 |
| Orthoptera | Tetrigidae | *Tetrix* | *Tetrix subulata* | NA | NA | NA | AY590161 | AY590171 | NA |
| Orthoptera | Acrididae | *Schistocerca* | *Schistocerca gregaria* | NA | EU203930 | MW962721 | AF232901 | AF145492 | M83966 |
| Orthoptera | Acrididae | *Locusta* | *Locusta migratoria* | KM853191 | KM853499 | AF370817 | EF546760 | JQ026101 | NA |
| Blattaria | Corydiidae | *Eupolyphaga* | *Eupolyphaga sinensis* | NA | MF286952 | NA | MF286820 | HQ615874 | MF287035 |
| Blattaria | Blattidae | *Blatta* | *Blatta orientalis* | FJ806323 | FJ806521 | AY521699 | DQ874031 | FJ806140 | MK798135 |
| Zygentoma | Lepidotrichidae | *Tricholepidion* | *Tricholepidion gertschi* | AF370789 | EU289816 | AF110863 | NA | AY555556 | NA |
| Microcoryphia | Machilidae | *Machilis* | *Machilis sp.* | AY338690 | AY338647 | AY521695 | NA | NA | NA |
| Mantophasmatodea | Mantophasmatidae | *Tyrannophasma* | *Tyrannophasma gladiator* | DQ457303 | DQ457338 | DQ457399 | DQ457230 | DQ457266 | DQ457369 |
| Mantophasmatodea | Mantophasmatidae | *Sclerophasma* | *Sclerophasma paresisensis* | DQ457302 | DQ457337 | AY521712 | NA | DQ457265 | NA |
